# Latent classes and progression of Mini-Mental State Examination scores in young-onset dementia: Data from the Swedish Dementia Register

**DOI:** 10.1177/13872877251376040

**Published:** 2025-09-11

**Authors:** Deborah Finkel, Fanny Kårelind, Steven H Zarit, Helle Wijk, Therése Bielsten, Linda Johansson

**Affiliations:** 1 Institute of Gerontology, School of Health and Welfare, Jönköping University, Jönköping, Sweden; 2 Center for Economic and Social Research and Department of Psychology, University of Southern California, Los Angeles, CA, USA; 3 Department of Human Development and Family Studies, Penn State University, University Park, PA, USA; 4 Institute of Health and Care Science, The Sahlgrenska Academy at Gothenburg University, Gothenburg, Sweden; 5 Department of Architecture and Civil Engineering, Chalmers University of Technology, Gothenburg, Sweden

**Keywords:** Alzheimer’s disease, longitudinal data analysis, progression, support, trajectory

## Abstract

**Background:**

Studies have shown significant heterogeneity in the longitudinal progression of dementias, including Alzheimer's disease. Growth mixture models have detected up to 4 classes that differ in both baseline Mini-Mental State Exam (MMSE) and rate of decline over time. Most analyses focus on adults over age 65 and investigate group differences in demographic and health variables.

**Objective:**

The current analysis focused on adults with young onset dementia (YOD) and examined the role of demographic and support variables in differentiating latent classes of longitudinal progression of cognitive status.

**Methods:**

Sample included 1025 adults (55% women) registered in the Swedish Dementia Register prior to age 65 with at least 3 registrations. Age at baseline was 38 to 64 (mean = 59.3, SD = 4.1); follow-up duration ranged from 1 to 12 years (mean = 4.6, SD = 2.0).

**Results:**

Growth mixture models identified 5 classes: high baseline MMSE and moderate decline over time (48.5%), intermediate baseline MMSE and moderate decline (34.5%), high baseline MMSE and steep decline (13.4%), low baseline and generally stable MMSE over time (2.6%), and high baseline with precipitous decline (1.0%). Latent classes differed in age at diagnosis, diagnostic categories, number of medications, and having home help services.

**Conclusions:**

Results highlight that YOD is just as heterogeneous as later onset dementia; therefore, it is vital that people with YOD get early diagnosis and a case manager to help identity and meet their individual needs.

## Introduction

The increasing proportion of older adults in the world population^1^ is associated with increasing prevalence of diseases and conditions associated with aging processes, including dementia.^2^ Research has focused on identifying protective factors that can reduce dementia risk and slow disease progression,^3^ and evidence suggests that the progression of dementia results from a combination of complex processes across multiple domains.^4^ The large heterogeneity in the progression of dementia that results from these multi-faceted processes provides an opportunity to identify factors associated with slower rates of progression, which in turn can contribute to improved understanding of prognoses and care needs.^5–7^ Given the several halted or modestly performing clinical drug trials for treatment of dementia,^8^ it is essential to develop treatments and support based on individual disease progression. Consequently, there has been a recent growth in studies aiming to identify distinct types of progression in dementia and then ascertain the factors associated with different patterns of progression. The majority of these studies focus on individuals with typical dementia onset (e.g., after age 65), with little attention paid to the possible heterogeneity of progression in individuals with young onset dementia (diagnosis prior to age 65).

In general, two approaches have been used to investigate patterns of dementia progression.^4^ First, many studies create groups based on diagnosis or baseline functioning and then follow these *a priori* defined groups over time to identify any differences in disease progression and predictive factors (e.g.,^9,10^). Second, studies have applied univariate or multivariate growth mixture models to longitudinal data to identify latent classes of individuals who show similar patterns of progression in outcome variables over time. Application of these models has varied across studies in multiple ways: estimation of linear^11–13^ or quadratic change trajectories, or both linear and quadratic^6^; use of preclinical or population-based samples^12,14^ versus samples with dementia diagnoses; dementia-specific outcomes variables such as Mini-Mental State Examination (MMSE) scores or neuropsychiatric symptoms^6,15^ versus more general measures such as memory performance^13^; length of follow-up (ranging from 2 to 12 years); and sample size, ranging from a few hundred to a few thousand. Not surprisingly, there has been considerable variety in results, even when two samples were compared within the same paper.^16^ The primary outcome of interest is the identification of latent classes of progression in functioning, and the number of latent classes identified has ranged from two to six. Generally, as reported in a recent review,^4^ researchers have identified latent classes with slower versus faster decline and often, although not always, groups with lower values on the outcome variable at intake demonstrate faster rates of decline.

Most studies investigating latent classes of dementia progression are consistent in the age of the sample. Even when the sample is identified as having mild cognitive impairment^15^ or is a population-based sample,^12^ the participants are generally over age 65 and generally the mean age of the sample is between 75 and 85. Few studies have investigated dementia progression in young onset dementia and most focused on comparing differences *between* young onset dementia and late onset dementia. In international consensus definitions, young onset dementia is defined by symptom onset before the age of 65.^17^ Some research indicates that individuals with young onset dementia show faster rates of decline in dementia symptomatology^18,19^ and are at a greater risk of mortality.^20^ Another study found no age differences in change in MMSE over time, but did report faster declines in instrumental activities of daily living in individuals with young onset dementia.^21^ To our knowledge, only two studies have investigated heterogeneity in dementia progression *within* groups with young onset dementia and both examined differences between *a priori* defined groups. Comparison of groups with high and low education indicated faster rates of decline in young onset dementia for individuals with more education.^22^ Different diagnostic categories also show different rates of decline over two years: individuals with Alzheimer's disease (AD) declined at twice the rate of individuals with vascular dementia or frontotemporal dementia.^23^

The aim of the current analysis was to apply growth mixture models to longitudinal data to investigate heterogeneity in dementia progression in individuals with young onset dementia. A key challenge to this approach is to identify longitudinal samples with sufficient size and length of follow up to support identification of multiple latent classes of disease progression.^24^ For these analyses, data for adults with dementia diagnosis prior to age 65 were extracted from the population-based Swedish Registry of Cognitive/Dementia Disorders, a nationally representative register. Annual follow-up for up to 12 years in a sample of 1025 individuals provided longitudinal assessment of cognitive status for use in identifying latent classes with similar patterns of dementia progression. We hypothesize that individuals with young onset dementia will show heterogeneity in disease progression that is similar to the heterogeneity identified in individuals with late onset dementia. Moreover, identified latent classes will differ in demographic and diagnostic characteristics.

## Methods

### Participants

This study examined baseline and longitudinal data from individuals under age 65 at the time of registration in the population-based Swedish Registry of Cognitive/Dementia Disorders (SveDem), covering data recorded from January 2009 to April 2022.^25,26^ Established in 2007, SveDem supports research and monitors guideline implementation, covering all memory clinics and 78% of primary care units in Sweden.^27,28^ The initial sample included 4458 individuals; however, baseline data were missing for 208 persons, leaving a sample of 4250. SveDem guidelines recommend at least annual follow-up of individuals. For the purposes of longitudinal analyses, only individuals with 3 or more registrations in SveDem were included in the final sample (N = 1025). The sample was 54.83% women and 100% European ancestry.

### Measures

Local users, typically nurses or physicians, entered relevant data into the SveDem registry. They relied on the patient´s medical records as the primary source of information. If information was not documented in the medical records, it was considered “not performed” in SveDem.^27^

*Baseline measures.* Number of medications, registered at baseline, was used as a proxy for general health. Missing values resulted in a sample of 989 with medication information. Support at baseline was registered for 5 forms of support: special housing, daycare, counselor, support for relatives, and home help service.^25,27^ Registrations for counselor, support for relatives and home help service were in dichotomous form: yes or no. Registrations for special housing were dichotomized to indicate normal housing versus any special housing (temporary care facility, care facility adapted for persons with dementia, care facility not adapted for person with dementia). Registrations for day care services were dichotomized to indicate no daycare versus any daycare (adapted for persons with dementia, adapted for person with early onset dementia, not adapted for person with dementia). Missing values resulted in N's ranging from 985 to 1023.

#### Longitudinal measures

The primary focus of the analyses was longitudinal registrations of MMSE scores.^29^ Baseline and follow-up diagnosis and global judgments of functioning (rated by staff) were also used in follow-up analyses. Diagnoses were determined by physicians using the International Classification of Diseases (ICD) classification system. Nine diagnostic categories were represented in the sample: AD, AD with vascular dementia, AD with Parkinson's disease, frontotemporal dementia, vascular dementia, Lewy body dementia, unspecific dementia, mild cognitive impairment (MCI), and other. The “other” diagnostic category could include dementia caused by Creutzfeldt-Jakobs disease, Huntington's disease, human immunodeficiency virus, dementia associated with Down syndrome, or alcohol-related dementia. According to the information provided by SveDem, a diagnosis of MCI was used only if the persons were expected to be diagnosed with AD and were medicated for it as well.^30^ For some analyses, diagnoses were dichotomized to create an AD variable: yes (AD, AD with vascular dementia, AD with Parkinson's disease) versus no (frontotemporal dementia, vascular dementia, Lewy body dementia, unspecific dementia, MCI, and other). At most follow-up registrations (approximately 95%), staff made a global judgment of any change in the functioning of the individual: worse functioning (−1), no change (0), or better functioning (1).

### Statistical methods

Statistical approach involved 3 steps. First, growth mixture models (GMM) were used to identify latent classes of longitudinal trajectories of MMSE in the sample. GMM are a multilevel modeling technique, similar to SEM and hierarchical linear growth modeling, that allows for the empirical identification of subgroups with homogenous trajectories from a large, heterogeneous sample with longitudinal data.^31^ The current analyses used time-based growth curve models to estimate the trajectories of change in MMSE since diagnosis (i.e., first registration in SveDem), centered at median follow-up time. Preliminary analyses indicated that a quadratic model provided a better fit to longitudinal change over time than a linear model (likelihood ratio test of difference in model fit = 24.30, df = 3, *p* < 0.01); therefore, the quadratic model was used in the GMM. For each class, intercept, linear change, and quadratic change over time were estimated, as well as the proportion of the sample in that class. Selection of the best model involves multiple factors including model convergence, examining Akaike Information Criterion (AIC), Bayesian Information Criterion (BIC), sample-sized adjusted BIC, likelihood ratio tests of nested models, entropy, and substantive considerations.^32^ GMM were fit using the hlme (heterogenous linear mixed models) function in R version 4.3.2.^33^

Second, analysis of variance and chi-square tests were used to examine differences between identified latent classes on demographic characteristics, diagnoses, and support measures. If the analysis of variance indicated significant mean differences between classes, post-hoc Tukey analyses of means were conducted to identify the differences. Given the large differences in class size and the skew in some of the variables, comparisons across classes were repeated using the nonparametric Kruskal-Wallis test: results were the same. Third, analysis of variance and chi-square tests were used to examine any change over time in diagnoses and global judgments within the identified latent classes. Class comparisons were conducted using SAS version 9.4.^34^

## Results

### Demographics

Demographic characteristics of the sample at first registration in SveDem (baseline) are presented in Table 1.

**Table 1.** Demographic characteristics of the sample at baseline.

Variable	N	Statistic	Range
Percent Female	1025	54.83%	
Mean Age (SD)	1025	59.26 (4.06)	38–64
Mean Number Registrations (SD)	1025	4.26 (1.56)	3–14
Mean Follow-up Interval (SD)	1025	1.09 (0.37)	0.30–3.14
Mean Years Follow-up (SD)	1025	4.55 (1.97)	0.90–12.08
Mean Number Medications (SD)	989	3.50 (2.80)	0–18
Mean MMSE (SD)	1025	23.02 (4.38)	5–30
Percent in Special Housing	1023	1.56%	
Percent in Daycare	1001	14.49%	
Percent with Counselor	1001	47.95%	
Percent Support for Relatives	985	74.92%	
Percent Home Help Services	1016	5.12%	
Diagnosis	1025		
Alzheimer's disease (AD)		66.74%	
AD with vascular dementia		3.90%	
AD with Parkinson's disease		1.66%	
Frontotemporal dementia		6.24%	
Vascular dementia		6.34%	
Lewy Body dementia		2.15%	
Unspecified dementia		9.17%	
Mild cognitive impairment		0.68%	
Other		3.12%	

Mean age of diagnosis was 59.26; however, age had significant negative skew, with 56.48% of the sample diagnosed between 60 and 64 and 29.95% of the sample diagnosed between 55 and 59. Mean number of registrations was 4.26 and only 9.56% of the sample had more than 6 registrations; number of registrations was limited by the window of observations for the data retrieved from SveDem (January 2009 to April 2022). Although the number of medications ranged from 0 (40 individuals) to 18 (1 individual), most of the sample (72.60%) took 4 or fewer medications. MMSE at baseline was fairly normally distributed, with some negative skew (−0.81): mean MMSE at baseline was 23.15 and the median value was 24. Using the dichotomized AD variable, 72.30% of the sample had AD, either alone or with an additional diagnosis.

### Growth mixture modeling

All models converged and the results of fitting five growth mixture models to the longitudinal MMSE data are presented in Table 2.

**Table 2.** Results of fitting the growth mixture model to longitudinal MMSE.

Model	Parameters	Log-Likelihood	AIC	BIC	SABIC	Entropy	Likelihood Ratio Test
1 class	10	−9653.12	19326.25	19375.57	19343.81	1.00	
2 classes	14	−9613.83	19255.66	19324.72	19280.25	0.90	39.29**
3 classes	18	−9583.63	19203.25	19292.04	19234.87	0.89	30.20**
4 classes	22	−9562.58	19169.15	19277.66	19207.79	0.74	21.05**
5 classes	26	−9550.06	19151.99	19280.36	19197.65	0.63	12.52*

AIC: Akaike Information Criterion; BIC: Bayesian Information Criterion; SABIC: sample-size adjusted BIC

* *p* < 0.05; ** *p* < 0.01

The likelihood ratio test compared the fit of each model to the previous model (i.e., model 2 versus model 1, model 3 versus model 2, etc.), to examine the value of adding estimation of an additional latent class. Comparison of model fit information resulted in mixed results: BIC was minimized for the 4-class model, but AIC and SABIC were minimized for the 5-class model. As expected, entropy was reduced with each additional latent class; however, all models produced an estimate of entropy greater than 0.60.^35^ Likelihood ratio comparisons indicated that each addition of an additional latent class resulted in a significant improvement of model fit to the data. The final class identified in the 5-class model constituted a significant 13.36% of the sample and represented an important distinction from Class 1, from which it was primarily drawn. As shown in the raw data trajectories presented in Supplemental Figure 1, Class 5 and Class 1 share a similar high MMSE at baseline; however, Class 1 shows modest declines in MMSE over time, whereas Class 5 shows steep decline in MMSE. Note that fitting a model with 6 classes resulted in poorer model fit statistics and class sizes less than 10. Therefore, relying on both model-fit statistics and substantive considerations, the 5-class model was chosen as the final model. Note that the analyses were repeated with a reduced sample that removed individuals with MCI (N = 7) and “other” (N = 32) diagnoses. The results were similar: AIC and SABIC were minimized in the 5-class model and BIC was minimized in the 4-class model. The same 5 classes were identified with similar posterior classification percentages.

**Figure 1.**
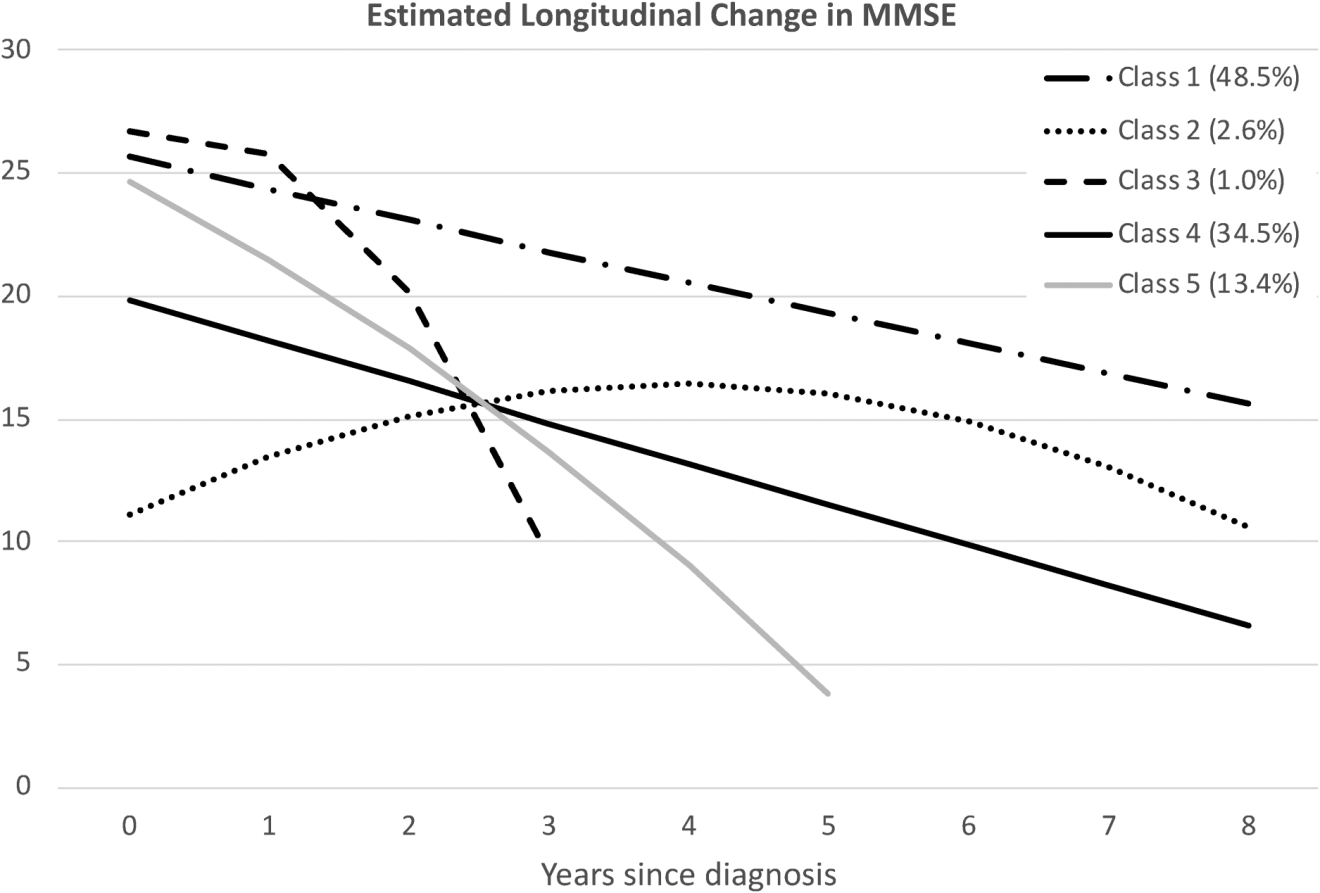
Estimated longitudinal trajectories for the classes identified in the 5-class growth mixture model. Percentage of the sample falling in each class is indicated in parentheses.

Parameter estimates from the 5-class model are presented in Supplemental Table 1. Longitudinal trajectories estimated from the parameter estimates are presented in Figure 1. Latent classes are numbered in the order in which they were identified in the growth mixture model; the percent of individuals in each class is presented in parenthesis. Members of the largest class (Class 1) had high MMSE values at baseline and modest linear decline over time: the quadratic term of the latent growth model was not significantly different from zero and MMSE score decreased by 1.2 points per year, on average. This trajectory is paralleled by the second largest class (Class 4). Although Class 4 had a baseline MMSE value approximately 5 points lower than class 1, on average, the rate of decline over time was similarly linear and modest. The third largest class (Class 5) had a high MMSE value at baseline, similar to Class 1, but showed a steep and accelerating rate of decline in MMSE over time (4.16 points per year). These three classes make up 96.39% of the sample. The two remaining classes (2 and 3) are quite small and have very distinct trajectories. Class 2 (N = 27) has a very low initial MMSE value, but values appear to increase and then decrease somewhat over time. Class 3 (N = 10) has a high initial MMSE value, similar to Classes 1 and 5, but estimates of linear and quadratic change were largest in Class 5, resulting in a precipitous drop in MMSE scores (5.72 points per year) after the first follow-up. The five estimated longitudinal trajectories in Figure 1 are very similar to the raw trajectories presented in Supplemental Figure 1.

### Class comparisons

Comparisons between the five identified latent classes on demographic, support, and diagnostic variables are reported in Table 3.

**Table 3.** Comparisons across latent classes identified by growth mixture model.

Variable	Class 1	Class 2	Class 3	Class 4	Class 5	Statistical Test
N	497	27	10	354	137	
Percent female	51.51	44.44	40.00	59.89	56.93	χ^2^ (df = 4) = 8.18
Mean age at baseline (SD)	59.41 (4.15)	57.59 (5.34)	60.30 (4.22)	58.88 (3.93)	59.93 (3.58)	*F*(4,1020) = 3.22 *
Mean number registrations (SD)	4.57 (1.73)	4.04 (1.56)	3.50 (0.71)	3.91 (1.32)	4.17 (1.33)	*F*(4,1020) = 10.51 **
Mean years follow-up (SD)	5.00 (2.08)	4.42 (1.83)	3.50 (0.86)	4.10 (1.83)	4.21 (1.65)	*F*(4,1020) = 13.43 **
Mean follow-up interval (SD)	1.13 (0.41)	1.11 (0.33)	1.01 (0.19)	1.06 (0.35)	1.02 (0.25)	*F*(4,1020) = 3.40 *
Mean number medications (SD)	3.56 (2.88)	4.19 (3.04)	2.50 (2.07)	3.68 (2.93)	2.79 (1.99)	*F*(4984) = 3.31 *
Mean MMSE at baseline (SD)	25.95 (2.21)	10.77 (2.61)	26.88 (2.42)	19.47 (2.34)	24.77 (2.30)	*F*(4,1020) = 6.18 **
Percent special housing	1.41	3.70	0.00	2.27	0.00	χ^2^ (df = 4) = 4.34
Percent daycare	11.93	14.81	30.00	17.39	15.04	χ^2^ (df = 4) = 6.88
Percent counselor	48.77	59.26	60.00	48.55	40.15	χ^2^ (df = 4) = 5.36
Percent support to relatives	73.60	73.08	77.78	78.57	70.68	χ^2^ (df = 4) = 4.19
Percent home help services	4.04	18.52	10.00	6.32	2.94	χ^2^ (df = 4) = 14.02 **
Percent AD diagnosis	63.78	62.96	50.00	74.22	89.05	χ^2^ (df = 4) = 38.64 **
Percent with change in diagnosis	15.45	3.70	20.00	10.48	9.49%	χ^2^ (df = 4) = 10.23 *

* *p* < 0.05; ** *p* < 0.01.

There were no significant differences between latent classes in percent female, although the chi-square test approached significant (*p* = 0.0853). There was a trend, then, for a greater proportion of women in Classes 4 and 5 and a greater proportion of men and Classes 2 and 3. The sex distribution was nearly equal in Class 1. There were no significant differences between latent classes on 4 of the 5 support variables; however, Class 2 was significantly more likely to have home help services at baseline (18.52%) than the other classes. There were significant differences in mean baseline age, and follow-up analyses indicated that mean age in Class 2 was significantly younger than mean age in Class 5. Classes 1, 3, 4, and 5 had mean baseline ages of 59 to 60, whereas mean age in Class 2 was 57.59. As expected, class differences in mean number of registrations and mean years follow-up were associated with estimated rate of decline for each class: steeper rates of decline were associated with fewer follow-up registrations. Post-hoc testing indicated that Class 1 had significantly more registrations and significantly longer follow-up than Class 5. Class 1 also had a significantly longer average interval between follow-up registration than Class 5, although follow-up interval was approximately 1 year for all 5 latent classes. Class 2 had the highest mean number of medications (4.19) compared with the other classes. Classes 1, 3, and 5 had similar baseline mean MMSE scores, which were significantly higher than mean MMSE scores in Classes 2 and 4. In fact, 27.5% of Class 1 had an MMSE at baseline of 28 or higher, as did 30% of Class 3 and 10.61% of Class 5. The relatively high MMSE scores (≥28) observed at the time of diagnosis among several individuals may reflect differences in diagnostic practices. In memory clinics, dementia diagnoses are typically based on a comprehensive clinical assessment that included neuropsychological testing, imaging, and functional evaluations, rather than relying solely on MMSE scores.

**Figure 2.**
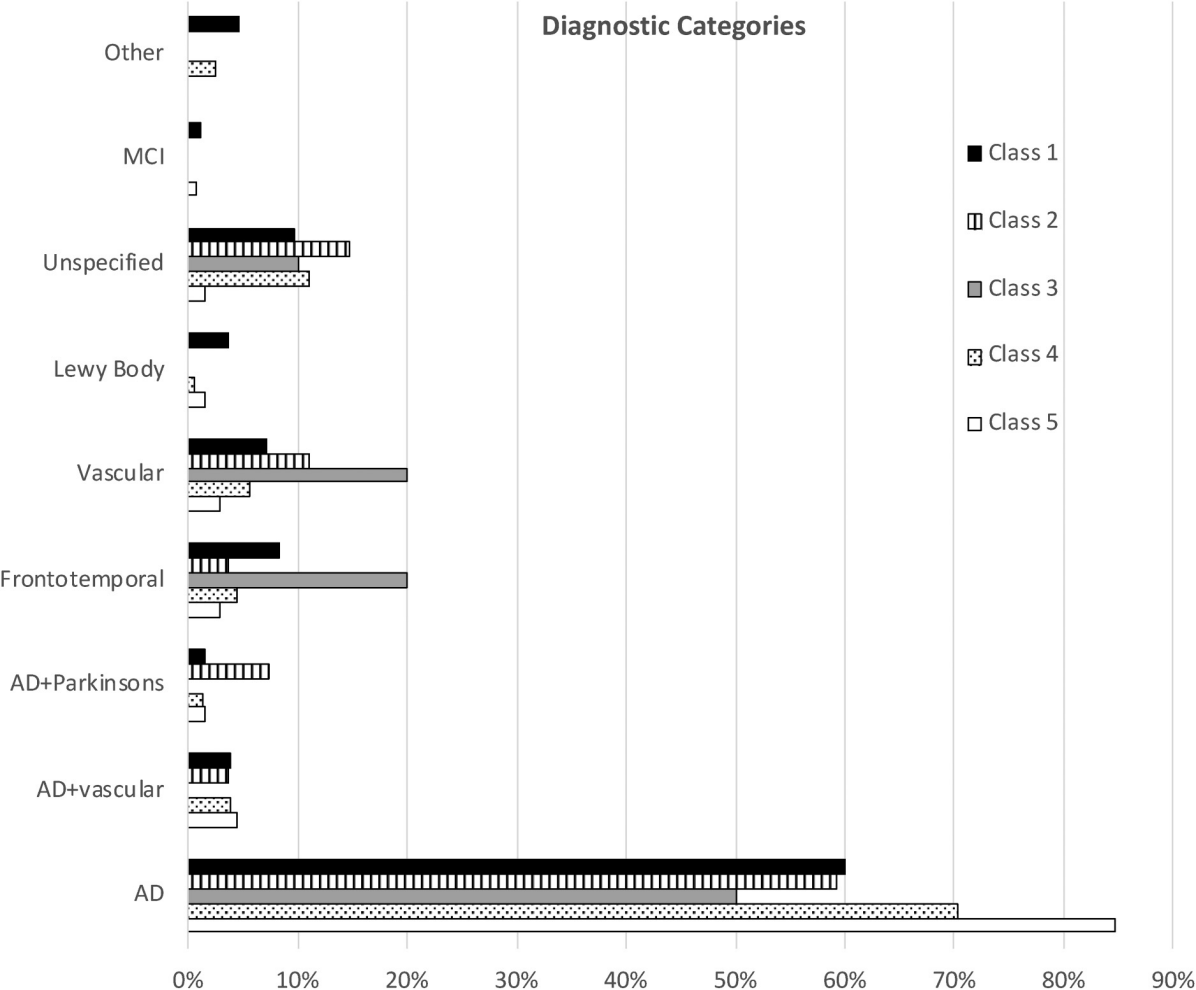
Percentage of in each diagnostic category in each latent class.

Class 5 had the highest proportion with any AD diagnosis (89.05%). Proportion of class members with each of the 9 possible diagnoses are presented in Figure 2. Differences in diagnostic categories across classes were significant (chi-square (df = 32) = 74.24, *p* < 0.01). Class 5 had the highest proportion with AD alone (84.67%). Class 3 had the lowest proportion with AD alone (50.00%) but had the highest proportions frontotemporal (20.00%) and vascular (20.00%) dementias. Class 2 had the highest proportions of AD with Parkinson's disease and unspecified dementia.

### Changes in diagnoses and global judgments

Although everyone in the sample has at least 3 registrations in SveDem, 85.35% of the sample has only one diagnosis registered in the database. Of the 150 individuals with more than one diagnosis registered in SveDem, 129 had a change in diagnosis registered after baseline. Most changes in diagnosis were for individuals who originally had an “unspecified” diagnosis: 33 of these 46 people had their diagnosis changed to another diagnostic category. For these 33 people, new diagnoses were distributed across all categories, although 23 of the new diagnoses were some form of AD. Note that in 5 cases a diagnosis of MCI was changed to a diagnosis of AD, which could reflect disease progression or refinement of diagnosis. The MCI diagnosis option was only added to the SveDem database in 2021. As a result, in 21 cases diagnosis changed to MCI from another diagnostic category: AD (9), vascular (4), frontotemporal (3), unspecified (3), and other (2). There was a significant difference across classes in percent with a change in diagnosis (chi-square (df = 4) = 10.23, *p* < 0.05); percent in each class with a change in registered diagnosis is indicated in the last row of Table 3. Details about changes in diagnoses in each class (for the 129 individuals with a change) are provided in Supplemental Table 2. Besides a reduction in “unspecified” diagnosis in most classes, there was no indication of particular trends for diagnostic changes in any class.

**Figure 3.**
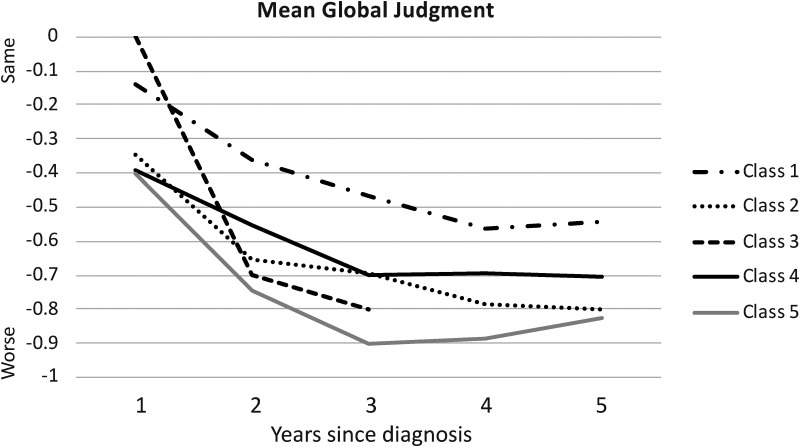
Longitudinal change in global judgment of functioning across latent classes.

Changes over time in global judgment, reflecting a holistic rating by care staff, were compared across latent classes at the first 5 follow-up registrations; sample sizes in some classes were greatly reduced after that. Changes in mean global judgment in each class are presented in Figure 3 and means and results of analyses of variance are reported in Supplemental Table 3. All analyses of variance testing differences in means among latent classes were significant. As shown in Figure 3, changes in global judgments over time were similar to, but not identical with, the estimated longitudinal trajectories for MMSE presented in Figure 1. After an initial decline in all latent classes, there was a decrease in the rate of decline in global judgment ratings, although this may have resulted in part from a “floor” effect due the limited range in values for the variable. Class 1 and Class 3 had similar ratings of “no change” at the first year after diagnosis, on average, but over time Class 1 showed the slowest decline in global judgment ratings and Class 3 showed the fastest decline. Classes 2, 4, and 5 all had mean global judgments at the first year after diagnosis of about −0.40; however, Class 5 showed more decline over time than the other two classes. By the third year after diagnosis, Class 3 and Class 5 had the worst mean global judgment ratings.

## Discussion

Application of growth mixture models to longitudinal MMSE data from the Swedish Registry of Cognitive/Dementia Disorders identified 5 latent classes of disease progression, indicating that young onset dementia is as heterogenous as late onset dementia in trajectories of decline in cognitive function. Although traditionally young onset dementia has been identified with steep decline,^18,19^ both fast and slow rates of change over time were identified. The largest latent class (Class 1) had high MMSE scores at baseline, on average, and a modest rate of linear decline over time (∼1 point per year). As a result, individuals in Class 1 had the longest mean follow-up period of 5 years. Decline in the second largest class (Class 4) paralleled Class 1, with a mean baseline MMSE score that was 5 points lower. These two classes accounted for a combined 83% of the sample; however, important variations in decline trajectories and group characteristics were identified in the remaining classes.

With 13.4% of the sample, Class 5 demonstrated both high baseline MMSE and steep and accelerating decline (∼4 points per year). This group had the highest rate (89%) of AD dementia diagnosis in the sample. Diagnosis of AD has changed dramatically since it was first identified, with a move from a focus on neuropsychological deficits to the increased use of biomarker indicators of dementia.^36^ The high initial functioning followed by rapid decline found in Class 5 may represent a typology of dementia that is most like the original definition of substantial progressive cognitive impairment. Moreover, some researchers propose a rapidly progressive AD subtype that is characterized by MMSE declines of 4 to 6 points per year.^37^ The proportion of cases that meet a criteria for rapid progression varies with sample and definition, of course, but the largest studies report 10–13% rapid progression dementia in their samples^38,39^; similar to the proportion identified in the current analyses.

The three largest latent classes identified for young onset dementia in the current analyses generally replicate trajectories detected in studies of dementia progression in late onset dementia. Depending on the number of latent classes identified, studies tend to report groups with slow and fast rates of decline^5,14^ or slow, intermediate, and fast progression.^6,12,40^ Investigations of late onset dementia have also identified latent classes that have similar MMSE values at baseline yet show significantly different rates of decline,^5,7^ like Class 1 and Class 5 in the current analysis. Between 8% and 23% of these samples demonstrated high initial MMSE followed by rapid disease progression, compared with 13% in Class 5 in the current analysis.

Two additional latent classes were identified in the current analysis: both were small with unique trajectories and both included a majority of men, unlike the other three latent classes. Moreover, individuals in Class 2 had the youngest mean age of diagnosis, another indication of how this group differed markedly from the other latent classes. Individuals in Class 2 demonstrated trajectories that were most different from the other classes: initial slight improvement followed by decline. Other investigators have also found evidence for at least initial improvement in MMSE scores in some classes.^11,15^ Cohen and colleagues (2024) reported that individuals demonstrating stable MMSE over time were less likely to have the *APOE* ε4 allele associated with AD and more likely to have experienced traumatic brain injury and comorbid neuropathology (such as Parkinson's disease). Similarly, individuals in Class 2 in the current analysis were less likely to have a diagnosis of AD alone and more likely to have a diagnosis of AD with Parkinson's disease (generally more common in men^41^) or an unspecified diagnosis. Individuals in this class were also significantly younger at baseline, had a higher average number of medications, and were more likely to already have home help at the point of dementia diagnosis. In other words, they were more likely to have comorbidities and poor health that necessitated care than those in the other classes. It is possible that the level of care provided as part of SveDem (daycare, support to relatives, counselor) resulted in a temporary improvement in functioning, or at least a stabilizing effect over the follow-up period. Another interpretation is that modest improvements in functioning resulted from a decrease in alcohol use after receiving increased support from the health care system. In SveDem, alcohol dementia (generally more common in men^42^) was generally included in the unspecified category,^25^ the diagnostic category of 15% of individuals in Class 2. Although the difference in sex distribution between the classes was not significant, the elevated number of men (55%) in Class 2 also supports the conclusion that even though the sample size was small, Class 2 identified a distinct pattern for young onset dementia.

The final latent class identified (Class 3) was the smallest (N = 10) and after an initial high level of functioning individuals in this class demonstrated the steepest rate of decline in both MMSE (nearly 6 points per year, on average) and in global judgments. Individuals in this class were least likely to have an AD diagnosis and most likely to have a diagnosis of frontotemporal or vascular dementia. Both frontotemporal dementia^43^ and vascular dementia^44^ are more common in men. Even though the difference in sex distribution between the classes was not significant, 60% of Class 3 were men, the highest proportion of men in any of the latent classes identified in this analysis. Leoutsakos and colleagues (2014) also reported a latent class that experienced dramatic declines in MMSE that made up 11% of their sample. Higher education was the only variable that reliably predicted membership in their Class 3, but SveDem does not include information about education so a direct comparison could not be made.

The type of variables available from SveDem is one of the primary limitations of the current analysis. The purpose of SveDem is to support quality of care for persons with dementia and it is not designed to support dementia research, per se. Therefore, variables included in the registry focus on quality of care (support, diagnosis, follow-up) and did not necessarily include variables that are of interest to researchers (e.g., education, socio-economic status, etc.). This limitation is balanced by the fact that SveDem is a nationally representative database with the goal to register every dementia case in Sweden and incorporates annual follow-up. Another limitation arises from the nature of diagnosis and care in young onset dementia. Because dementia is not an expected diagnosis prior to age 65 and individuals with young onset dementia may have clinical presentations that differ from late onset dementia, they can experience delays in diagnosis.^45^ As a result, differences in rates of decline could result because individuals were identified at different points in the disease process (i.e., early in the disorder versus late); however, dementia trajectories identified in the current analysis are similar to trajectories reported in the literature for late onset dementia. Finally, the number of classes identified in growth mixture models will depend on the sample and on modeling decisions. Opting for more latent classes results in identifying more heterogeneity, but again, the results found here for young onset dementia generally replicated results reported for late onset dementia indicating faster, intermediate, and slower rates of decline.

In summary, then, the current analysis represents the first application of growth mixture models to the identification of latent classes of disease progression in younger onset dementia. It identified a similar degree of heterogeneity in disease progress as is found in late onset dementia. However, individuals with young onset dementia can face challenges unique to their position in the lifecourse, such as reduced employment resulting in financial strain, parenting challenges, and the stigma associated with symptoms and diagnosis not typical for their age.^46,47^ Support services are rarely designed specifically for individuals experiencing these challenges, resulting in feelings of neglect or lack of support by health services.^48^ These situations underscore the need for early and accurate diagnosis of young onset dementia to help identify and meet their individual needs. Moreover, identification of factors or diagnoses associated with slower rates of decline in young onset dementia, as in late onset dementia, can enhance understanding of progression and support increased responsiveness to individual care needs.^5–7^

## Supplemental Material

sj-docx-1-alz-10.1177_13872877251376040 - Supplemental material for Latent classes and progression of Mini-Mental State Examination scores in young-onset dementia: Data from the Swedish Dementia Register Supplemental material, sj-docx-1-alz-10.1177_13872877251376040 for Latent classes and progression of Mini-Mental State Examination scores in young-onset dementia: Data from the Swedish Dementia Register by Deborah Finkel, Fanny Kårelind, Steven H Zarit, Helle Wijk, Therése Bielsten and Linda Johansson in Journal of Alzheimer's Disease
