# Supplementary material for: Latent classes and progression of Mini-Mental State Examination scores in young-onset dementia: Data from the Swedish Dementia Register
Source: J Alzheimers Dis. 2025 Sep 11;108(1):262–72. doi: 10.1177/13872877251376040 (PMC12929668; doi:10.1177/13872877251376040)
Supplement: sj-docx-1-alz-10.1177_13872877251376040 - Supplemental material for Latent classes and progression of Mini-Mental State Examination scores in young-onset dementia: Data from the Swedish Dementia Register [file sj-docx-1-alz-10.1177_13872877251376040.docx]

**Supplemental Material**

**Latent classes and progression of Mini-Mental State Examination scores in young-onset dementia: Data from the Swedish Dementia Register**

**Supplemental Table 1.** Parameter estimates from the 5-class growth mixture model

| Class | N in class | Intercept (SE) | Linear (SE) | Quadratic (SE) |
| --- | --- | --- | --- | --- |
| 1 | 497 | 22.58 (0.61)** | -1.28 (0.15)** | 0.01 (0.03) |
| 2 | 27 | 15.61 (1.48)** | 1.05 (0.44)* | -0.35 (0.16)* |
| 3 | 10 | 16.55 (2.09)** | -10.05 (1.04)** | -2.43 (0.37)** |
| 4 | 354 | 15.85 (0.53)** | -1.66 (0.20)** | 0.00 (0.04) |
| 5 | 137 | 16.29 (0.89)** | -4.10 (0.28)** | -0.25 (0.08)** |

**p* < 0.05, ***p* < 0.01

**Supplemental Table 2.** Changes in diagnosis in each latent class, for the 129 individuals for whom diagnosis changed.

| **First Diagnosis** | **Last Diagnosis** | | | | | | | | |
| --- | --- | --- | --- | --- | --- | --- | --- | --- | --- |
|  | AD | AD/VaD | AD/PD | FTD | VaD | LBD | Uns | MCI | Other |
| **CLASS 1** | | | | | | | | | |
| AD | 0 | 1 | 1 | 1 | 1 | 2 | 4 | 6 | 1 |
| AD/VaD | 2 | 0 | 0 | 0 | 0 | 0 | 0 | 0 | 1 |
| AD/PD | 0 | 0 | 0 | 0 | 1 | 3 | 0 | 0 | 0 |
| FTD | 1 | 0 | 0 | 0 | 0 | 0 | 0 | 3 | 2 |
| VaD | 0 | 0 | 0 | 0 | 0 | 0 | 1 | 3 | 1 |
| LBD | 1 | 0 | 0 | 0 | 1 | 0 | 0 | 0 | 0 |
| Uns | 8 | 2 | 0 | 5 | 5 | 3 | 0 | 3 | 2 |
| MCI | 4 | 0 | 0 | 0 | 0 | 0 | 0 | 0 | 0 |
| Other | 0 | 0 | 1 | 0 | 2 | 1 | 1 | 2 | 0 |
| **CLASS 2** | | | | | | | | | |
| 1 individual diagnosis changed from “Unspecified” to AD | | | | | | | | | |
| **CLASS 3** | | | | | | | | | |
| 1 individual diagnosis changed from VaD to AD/VaD | | | | | | | | | |
| 1 individual diagnosis changed from VaD to MCI | | | | | | | | | |
| **CLASS 4** | | | | | | | | | |
| AD | 0 | 2 | 0 | 1 | 0 | 1 | 1 | 3 | 0 |
| AD/VaD | 0 | 0 | 0 | 0 | 1 | 0 | 1 | 0 | 0 |
| AD/PD | 0 | 0 | 0 | 0 | 0 | 1 | 1 | 0 | 0 |
| FTD | 1 | 0 | 0 | 0 | 0 | 0 | 0 | 0 | 2 |
| VaD | 0 | 1 | 0 | 0 | 0 | 1 | 1 | 0 | 1 |
| LBD | 0 | 0 | 0 | 0 | 0 | 0 | 0 | 0 | 0 |
| Uns | 0 | 0 | 0 | 0 | 0 | 0 | 0 | 0 | 0 |
| MCI | 0 | 0 | 0 | 0 | 0 | 0 | 0 | 0 | 0 |
| Other | 3 | 0 | 0 | 1 | 0 | 0 | 0 | 0 | 0 |
| **CLASS 5** | | | | | | | | | |
| AD | 0 | 2 | 0 | 1 | 1 | 1 | 0 | 0 | 0 |
| AD/VaD | 1 | 0 | 0 | 0 | 0 | 0 | 0 | 0 | 0 |
| AD/PD | 0 | 0 | 0 | 0 | 0 | 1 | 0 | 0 | 0 |
| FTD | 0 | 0 | 0 | 0 | 0 | 0 | 0 | 0 | 0 |
| VaD | 0 | 0 | 0 | 1 | 0 | 0 | 1 | 0 | 0 |
| LBD | 0 | 0 | 0 | 0 | 0 | 0 | 0 | 0 | 1 |
| Uns | 1 | 0 | 0 | 1 | 0 | 0 | 0 | 0 | 0 |
| MCI | 1 | 0 | 0 | 0 | 0 | 0 | 0 | 0 | 0 |
| Other | 0 | 0 | 0 | 0 | 0 | 0 | 0 | 0 | 0 |

AD: Alzheimer’s disease; AD/VaD: AD with vascular dementia; AD/PD: AD with Parkinson’s disease; FTD: frontotemporal dementia; VaD: vascular dementia; LBD: Lewy body dementia; Uns: Unspecified; MCI: mild cognitive impairment

**Supplemental Table 3.** Mean global judgment values in each latent class over time.

| Class | Follow-up 1 | | Follow-up 2 | | Follow-up 3 | | Follow-up 4 | | Follow-up 5 | |
| --- | --- | --- | --- | --- | --- | --- | --- | --- | --- | --- |
|  | N | Mean (SD) | N | Mean (SD) | N | Mean (SD) | N | Mean (SD) | N | Mean (SD) |
| 1 | 464 | -0.14 (0.66) | 477 | -0.36 (0.61) | 475 | -0.47 (0.62) | 324 | -0.56 (0.57) | 193 | -0.54 (0.54) |
| 2 | 23 | -0.35 (0.78) | 26 | -0.65 (0.63) | 26 | -0.69 (0.62) | 14 | -0.79 (0.58) | 5 | -0.80 (0.45) |
| 3 | 10 | 0.00 (0.67) | 10 | -0.70 (0.48) | 10 | -0.80 (0.43) | 4 | -0.75 (0.50) | -- | -- |
| 4 | 340 | -0.39 (0.66) | 343 | -0.55 (0.62) | 343 | -0.70 (0.52) | 166 | -0.69 (0.51) | 78 | -0.71 (0.56) |
| 5 | 134 | -0.40 (0.60) | 130 | -0.75 (0.45) | 133 | -0.90 (0.35) | 81 | -0.89 (-.32) | 40 | -0.83 (0.45) |
| ANOVA | *F*(4,966)=9.50** | | *F*(4,981)=13.38** | | *F*(4,982)=19.55** | | *F*(4,584)=7.06** | | *F*(3,312)=4.13** | |

**p* < 0.05; ***p* < 0.01


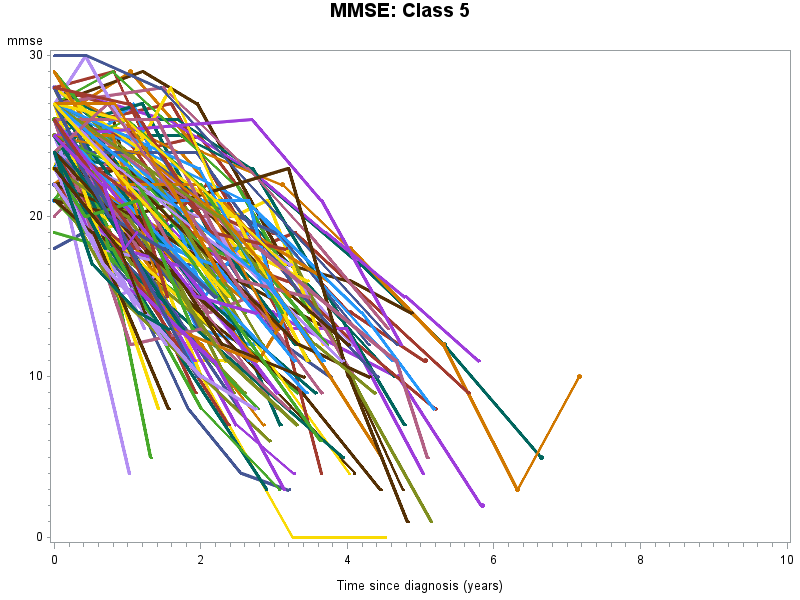

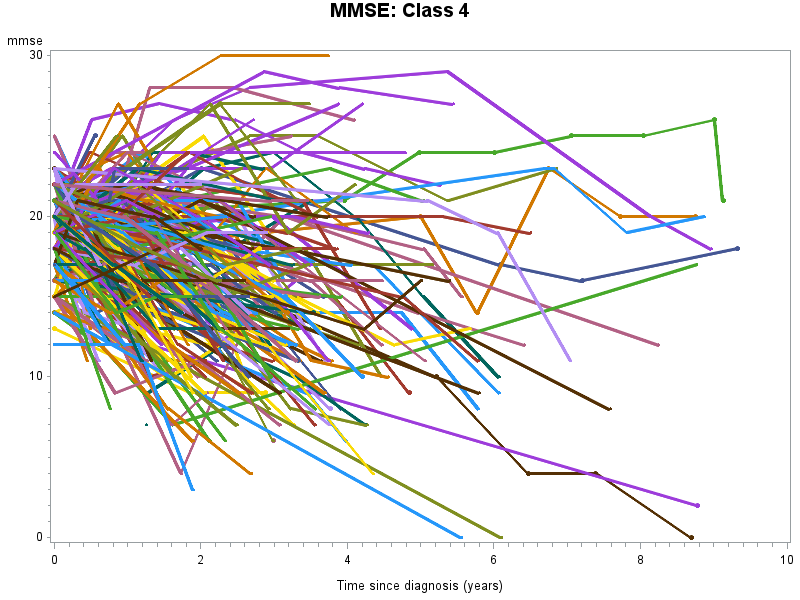

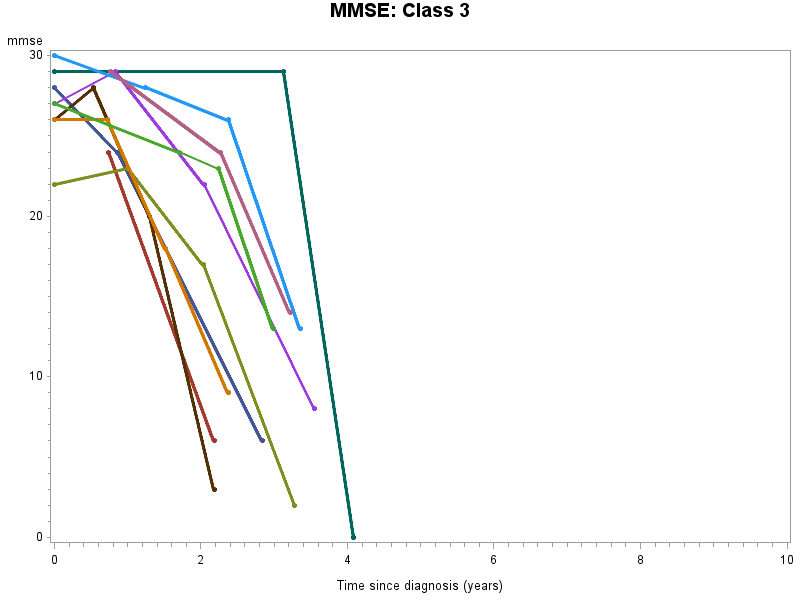

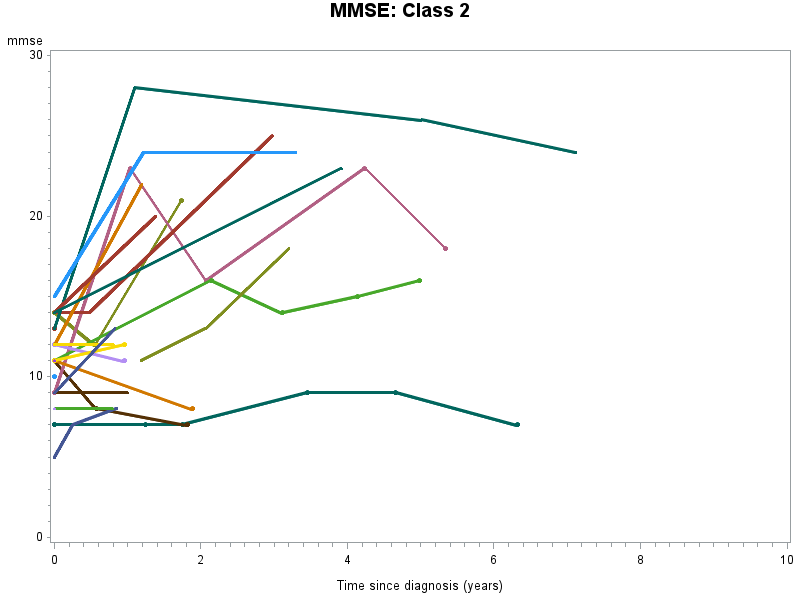

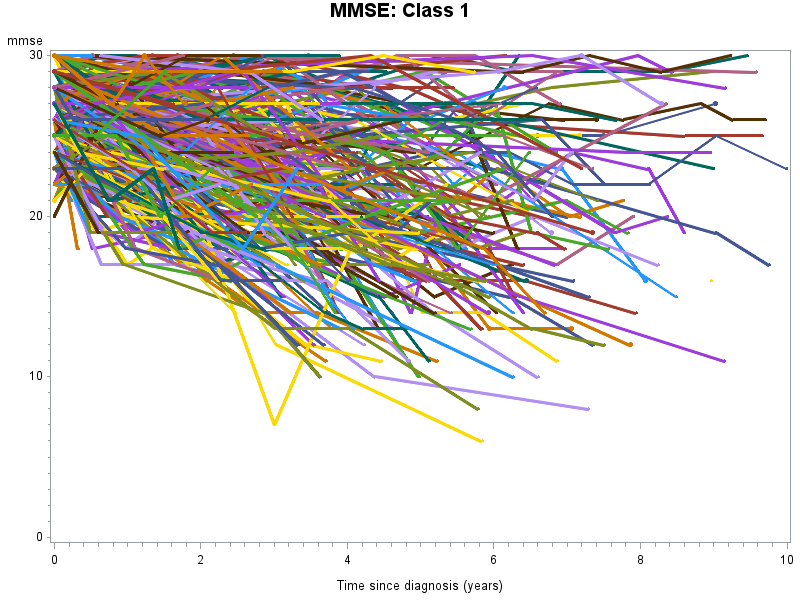


**Supplemental Figure 1.** Raw data longitudinal trajectories in each latent class.
